# Belimumab use during pregnancy: Interim results of the belimumab pregnancy registry

**DOI:** 10.1002/bdr2.2091

**Published:** 2022-09-30

**Authors:** Patricia Juliao, Keele Wurst, Jeanne M. Pimenta, Kim Gemzoe, Helain Landy, M. Anthony Moody, Hugh Tilson, Deborah Covington, Tammy Moore, Rebecca Marino, Jennifer Gilbride, Andrew Liu, Paige Meizlik, Michelle Petri

**Affiliations:** ^1^ Vaccines GSK Collegeville Pennsylvania USA; ^2^ Epidemiology GSK Research Triangle Park North Carolina USA; ^3^ Epidemiology GSK Uxbridge Middlesex United Kingdom; ^4^ Value Evidence and Outcomes GSK Stevenage Hertfordshire United Kingdom; ^5^ Maternal And Fetal Medicine Georgetown University Medical Center Washington DC USA; ^6^ Department of Obstetrics and Gynecology MedStar Georgetown University Hospital Washington DC USA; ^7^ Department of Pediatrics Duke University School of Medicine Durham North Carolina USA; ^8^ Department of Epidemiology University of North Carolina Gillings School of Global Public Health Chapel Hill North Carolina USA; ^9^ Real‐World Evidence Evidera Wilmington North Carolina USA; ^10^ PPD Wilmington North Carolina USA; ^11^ US Case Management Group GSK Research Triangle Park North Carolina USA; ^12^ Statistics GSK Stevenage Hertfordshire United Kingdom; ^13^ Global Clinical Safety and Pharmacovigilance GSK Uxbridge Middlesex United Kingdom; ^14^ Immunoinflammation GSK Collegeville Pennsylvania USA; ^15^ Rheumatology Johns Hopkins University School of Medicine Baltimore Maryland USA

**Keywords:** belimumab, pregnancy, major birth defects, systemic lupus erythematosus

## Abstract

**Background:**

Belimumab is approved for active, autoantibody‐positive systemic lupus erythematosus (SLE) and lupus nephritis, but limited data exist regarding its use in pregnancy. The Belimumab Pregnancy Registry (BPR, GSK Study BEL114256; NCT01532310) was created to evaluate pregnancy and infant outcomes following belimumab exposure.

**Methods:**

Individuals with SLE exposed to belimumab from 4 months before and/or during pregnancy can enroll into the BPR. The primary outcome is major birth defects; secondary outcomes include miscarriages, stillbirths, elective termination, pre‐term birth, neonatal death, small for gestational age, and adverse infant outcomes during the first year of life. Belimumab exposure timing, concomitant medications, and other potential confounding factors are also collected. Data up to March 8, 2021, are reported descriptively.

**Results:**

From an expected sample size target of 500 prospective pregnancies with a known outcome, only 55 were enrolled in the study. Among these, two pregnancy losses and 53 pregnancies with a live birth outcome were reported. Ten of the 53 live birth pregnancies resulted in a major birth defect. Ten pregnancies were enrolled after the pregnancy outcome occurred and were examined retrospectively (four live births with no defects, four miscarriages, and two elective terminations). There was no indication or pattern of birth defects associated with belimumab.

**Conclusions:**

Low recruitment numbers for the BPR and incomplete information limit the conclusions regarding belimumab exposure during pregnancy. There was no pattern or common mechanism of birth defects associated with belimumab within the BPR data.

## 1 INTRODUCTION

Systemic lupus erythematosus (SLE) is a chronic multisystem autoimmune disease that predominantly affects females of childbearing age (Naseri et al., 2018; Tsokos, 2011). Pregnant individuals with SLE have a higher risk than the general population or those without SLE for maternal morbidity and mortality, premature delivery, intrauterine growth restriction, preeclampsia, pregnancy loss, congenital malformation (Bundhun, Soogund, & Huang, 2017; Nili, McLeod, O'Connell, Sutton, & McMillan, 2013; Rowaiee, 2019; Vinet et al., 2015; Wallenius, Salvesen, Daltveit, & Skomsvoll, 2014), and neonatal lupus syndrome, the latter owing to transplacental passage of autoantibodies (Chakravarty, Nelson, & Krishnan, 2006; Clowse, Jamison, Myers, & James, 2008; Ruiz‐Irastorza & Khamashta, 2011; Tani et al., 2021). SLE manifestations may be difficult to distinguish from other pregnancy‐associated changes or complications not related to SLE (Naseri et al., 2018). In addition, several SLE treatments, such as cyclophosphamide, mycophenolic acid, leflunomide, and methotrexate, are known or suspected teratogens (American College of Rheumatology, 2020; Petri, 2020; Taulaigo et al., 2021). Although evidence suggests that favorable pregnancy outcomes can be achieved in SLE, particularly if pregnancy is planned during a period of remission, pregnancy has historically been discouraged in affected patients (Tani et al., 2021; Taulaigo et al., 2021). Disease severity and medications prescribed for SLE can contribute to the increased risk of adverse pregnancy outcomes associated with SLE.

Belimumab is a human immunoglobulin (Ig) G1 monoclonal antibody that targets B lymphocyte stimulator (BLyS; also known as B‐cell‐activating factor) to inhibit survival and differentiation of autoreactive B cells (Baker et al., 2003; GSK, 2022). In a cynomolgus monkey study, belimumab was detected in the fetal cord blood and amniotic fluid, indicating that the drug can cross the placental barrier (Auyeung‐Kim, Devalaraja, Migone, Cai, & Chellman, 2009; GSK, 2022). Further, this toxicology study did not show evidence of fetal harm after administration of intravenous (IV) belimumab with exposures approximately 9–20‐fold the maximum recommended human dose (Auyeung‐Kim et al., 2009; GSK, 2022). Belimumab is approved as an add‐on therapy in patients over 5 years of age with active, autoantibody‐positive SLE or active lupus nephritis who are receiving standard therapy (GSK, 2022). In Phase 3 trials, treatment with IV or subcutaneous belimumab plus standard therapy was effective and well tolerated in patients with SLE (Furie et al., 2011; Navarra et al., 2011; Stohl et al., 2017; Zhang et al., 2018). However, as in most clinical Phase 3 SLE trials (Taulaigo et al., 2021), pregnant individuals were excluded; consequently, available data on the use of belimumab during human pregnancy are limited.

Based on the lack of data, the use of belimumab during pregnancy should be considered only after a benefit‐risk assessment for the mother and fetus (European Medicines Agency, 2021; GSK, 2022). The European Alliance of Associations for Rheumatology and the British Society for Rheumatology guidelines generally advise caution for treatment with belimumab during pregnancy, whereas the American College of Rheumatology recommend discontinuation in those who become pregnant (Andreoli et al., 2017; Flint et al., 2016; Sammaritano et al., 2020). Thus, additional data on the effect of belimumab on pregnancy outcomes are needed, particularly as SLE predominantly affects women of childbearing age (Tani et al., 2021; Taulaigo et al., 2021).

Pregnancy registries are one approach for the collection of additional, post‐approval pregnancy safety data for a drug of interest (Food and Drug Administration, 2019), and have been established for several antirheumatic drugs (Taulaigo et al., 2021). The Belimumab Pregnancy Registry (BPR; GSK Study BEL114256; NCT01532310) was initiated in 2012 as a post‐authorization commitment study to the European Medicines Agency and the United States Food and Drug Administration. The overall aim of the registry was to evaluate pregnancy and infant outcomes following belimumab exposure. All pregnancies occurring since belimumab became commercially available in 2012 were eligible to participate in the BPR (Food and Drug Administration, 2019; Taulaigo et al., 2021).

Since its initiation in 2012, the registry has been unable to obtain a sufficient sample size for adequate precision estimates due to extremely low enrollment compared with the original targeted number. Although the study is not powered to provide definitive data, this manuscript provides descriptive results on belimumab exposure in pregnancy, infant outcomes, and describes the biases and limitations within the study design.

## 2 METHODS

### 2.1 Study design

The BPR (GSK Study BEL114256; NCT01532310) is an international, self‐enrollment cohort study collecting data from SLE pregnancies exposed to commercially available belimumab across Austria, Belgium, Canada, France, Germany, Israel, Italy, Portugal, Slovakia, Spain, Sweden, Switzerland, and the United States. Exposure was defined as at least one dose of belimumab administered 4 months prior to and/or during pregnancy. Confirmation of exposure was obtained by the prescribing healthcare provider (HCP) with information on gestational timing, route of administration, dose, and dates of exposure. Live‐born infants were followed up at 4 and 12 months.

Institutional Review Board (IRB)/Ethics Committee (EC) approval was obtained first in the US and Canada from the Western Institution Review Board on July 2012 and thereafter by the sites' IRB/EC within each participating country in Europe. Participant informed consent was required for participation in this pregnancy registry. Under applicable regulations in the United States and several European countries, written informed consent was waived, and verbal informed consent was allowed. Informed consent was obtained by the registry coordinating center staff, the principal investigator, or the participant's HCP following review of the Participant Information Sheet Consent Form.

### 2.2 Patient population and data collection

Individuals with SLE and an active pregnancy at time of enrollment who were exposed to commercially available belimumab from 4 months prior to and/or during pregnancy were enrolled prospectively into the BPR.

Data from belimumab‐exposed pregnancies that completed (including pregnancy loss or live birth) prior to enrollment were collected separately in a retrospective cohort to evaluate for pregnancy outcome patterns, rather than calculation of pregnancy outcome proportions, as this cohort would be subject to biases.

A participant was considered evaluable and included in the study if the data submitted or confirmed by an HCP met the minimum criteria for a registry report, which included: confirmed belimumab exposure, sufficient information to classify the pregnancy as prospective or retrospective, the pregnancy outcome, contact information for follow‐up and consent from the pregnant individual to participate in the registry.

Potentially eligible pregnant individuals could self‐enroll into the registry or were referred into the registry by their HCPs. As this registry was strictly observational, the schedule of office visits and all treatment regimens were determined by the treating HCP. The registry collected data from appropriate members of the participants' healthcare team. Data regarding participants' SLE disease severity was sought from the belimumab prescriber. Data regarding the pregnancy and pregnancy outcome was primarily sought from the obstetric HCP, and data on live‐born infants was sought from the pediatric HCP. Data were also accepted from other HCPs and parents in an established data hierarchy to maximize data collection. All data were collected on case report forms, which were sent to the various reporters at study registration, second trimester, pregnancy outcome, and 4 and 12 months of age for live‐born infants. The data included clinical data that were routinely documented in medical records. Medical records were not requested by the registry; however, the HCPs were encouraged to extract data from medical records to minimize recall bias.

Data were collected from the HCPs at study enrollment, at the end of the second trimester, and at pregnancy outcome (delivery or pregnancy loss). Information regarding timing of belimumab exposure, SLE disease severity, concomitant medications, and potential cofounders (age, race/ethnicity, comorbidities, prenatal tests, maternal risk factors, and pregnancy/general health history) were collected. The primary objective of the BPR was to evaluate major birth defects, assessed using the Metropolitan Atlanta Congenital Defects Program (MACDP) criteria from the US Center for Disease Control and Prevention (Centers for Disease Control and Prevention, 2019), and/or the European Surveillance of Congenital Anomalies (EUROCAT) criteria (EUROCAT, 2021) as individuals from North America and Europe were eligible to participate in the study. Specifically, for the MACDP criteria within the BPR, major birth defects were defined as any major structural or chromosomal defect or combination of at least two conditional defects (defined as conditions that appear in the MACDP Exclusion List) in live‐born infants, stillbirths, or pregnancy losses of any gestational age (including outcomes prior to 20 weeks gestational age or weighing <500 g). Per protocol, single defects that were excluded in MACDP were not considered major birth defect cases in the BPR. Potential birth defect cases initially reported by HCPs were reviewed by the study birth defect evaluator (BDE) to determine if it was a major birth defect outcome using the MACDP criteria and to assess the timing of belimumab exposure relative to the development of the observed major birth defect.

Secondary pregnancy outcomes were defined as: miscarriage (fetal death or expulsion of products of conception prior to 20 weeks gestation); molar pregnancy (a conception that results in a gestational trophoblastic tumor); ectopic pregnancy (implantation of a conception outside of the uterus); elective termination; stillbirth (fetal death at ≥20 weeks gestation, or if gestation is unknown, fetal weight ≥ 500 g); live birth (birth of living fetus at ≥20 weeks gestation, or if gestation is unknown, fetal weight ≥ 500 g); and combined pregnancy loss (pregnancy with either miscarriage, molar/ectopic pregnancy, or stillbirth outcome).

Secondary infant outcomes were defined as: pre‐term birth (infant born at gestational age < 37 weeks); neonatal death (infant who expired within 28 days of live birth), small for gestational age (SGA) for infant birth weight < 10th percentile for gestational age (assessed using the International Fetal and Newborn Growth Consortium for the 21st Century [INTERGROWTH‐21st Project]) (Villar et al., 2014), and the American College of Obstetricians and Gynecologists (ACOG) (Alexander, Himes, Kaufman, Mor, & Kogan, 1996); infant infection/fever (“any infection or fever of unknown origin” or “of known infectious etiology that occurs through three months of age”); and developmental milestones (provided by pediatrician at 4 and 12 months for the five main areas of development: gross motor, fine motor, cognitive, socio‐emotional, and language).

On an annual basis, a BPR‐specific Scientific Advisory Committee comprising experts in SLE, pediatrics, and obstetrics, and including the BDE, performed an independent review of the data collected, including review and classification of reported birth defects and review and interpretation of the cumulative data.

### 2.3 Data analysis

The registry aimed to enroll approximately 500 individuals with prospective pregnancies to assess the primary outcome of the study (major birth defects). This sample size would have resulted in approximately 350 live births. As no data on the background rates of major birth defects in the SLE population were known at the time of study development, the general population background rate of 2.78% (Correa et al., 2007) was used to calculate the sample size of 350 live births needed to provide a precision of ±1.89% or less around the point estimate (95% confidence interval [CI]: 1.32–5.09%). Given the challenges in enrolling this number of live births, the actual sample size was underpowered to statistically evaluate major birth defects as originally planned. Thus, descriptive analyses were conducted on all primary and secondary outcomes.

Gestational timing exposure was characterized as: preconception (4 months prior to date of conception), first trimester (the day after date of conception through week 13), second trimester (week 14 through week 27 after date of conception), and third trimester (week 28 or greater after date of conception). Date of conception was calculated using the estimated delivery date minus 266 days or the date of the last menstrual period plus 14 days.

Overall enrollment, demographic, and baseline characteristics for evaluable participants between July 16, 2012 and March 8, 2021 are summarized descriptively. The presence of variables that may potentially bias the association between exposure and the study outcomes (i.e., potential confounders) were also summarized descriptively. For the primary outcome, the number of infants with a major birth defect is reported. In addition, clinical characteristics of the major birth defects are also reported along with pregnancy exposure timing and potential confounders. For the secondary pregnancy outcomes (miscarriage, molar and ectopic pregnancies, elective termination, stillbirth, live birth, and pregnancy loss), the number of pregnancies with a confirmed outcome are reported. For the secondary infant outcomes (pre‐term, neonatal death, SGA, development milestone delays, and serious/clinical infections), the number of infants with confirmed outcomes regardless of pregnancies with multiple gestations are included.

Ad hoc 95% CIs for the proportion of live birth pregnancies with major birth defects or pregnancy loss were calculated using the Wald method (simple asymptotic) without continuity.

## 3 RESULTS

### 3.1 Registry enrollment

**FIGURE 1:**
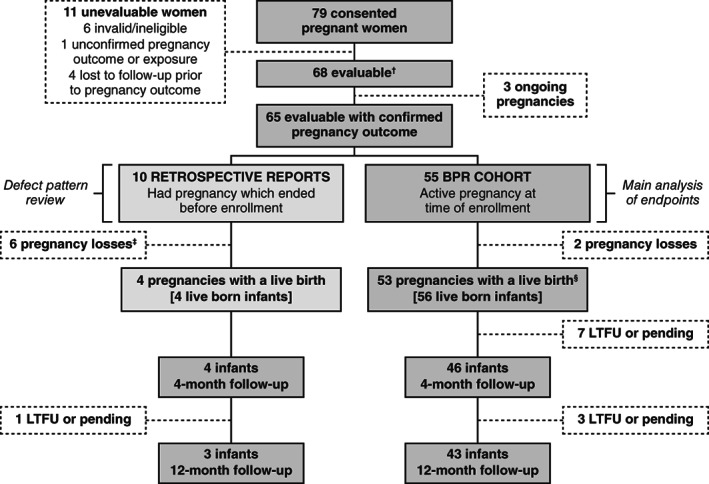
BPR study population and enrollment attrition results. BPR, Belimumab Pregnancy Registry (GSK Study BEL114256; NCT01532310); LTFU, lost to follow up. ^†^A participant was considered evaluable and included in the study if the data submitted or confirmed by an HCP met the minimum criteria for a registry report, which included: confirmed belimumab exposure, sufficient information to classify the pregnancy as prospective or retrospective, the pregnancy outcome, contact information for follow‐up and consent from the pregnant individual to participate in the registry; ^‡^Includes two elective terminations; ^§^three pregnancies with twins.

A total of 79 pregnant individuals consented to be included in the BPR, with 68 (86%) individuals considered evaluable, and 11 (14%) individuals considered unevaluable (Figure 1). Of the 68 evaluable individuals, three (4%) had ongoing pregnancies and 65 (96%) had confirmed pregnancy outcomes at data cutoff. The 65 evaluable individuals with confirmed outcomes ranged in age from 21 to 42 years (mean 31.7 years) and were recruited from six countries: United States (*n* = 51, 78%), Canada (*n* = 4, 6%), France (*n* = 3, 5%), Spain (*n* = 3, 5%), Austria (*n* = 2, 3%), and Germany (*n* = 2, 3%); no individuals were enrolled from the seven other countries in which the BPR was open. Among the 65 with confirmed outcomes, 55 (85%) individuals were pregnant at the time of enrollment and were prospectively enrolled into the BPR cohort to assess the main outcomes of the study. Of these 55 individuals, 41 (75%) had prenatal testing prior to enrollment (e.g., ultrasound, amniocentesis, maternal serum alpha‐fetoprotein test, Quad Screen, chorionic villus sampling, and other tests, as available), five (9%) had unknown prenatal testing status, and nine (16%) had no prenatal testing prior to enrollment (Table 1). The 10/65 (15%) individuals with completed pregnancies (such as live birth or pregnancy loss) prior to enrollment were included in the retrospective cohort to assess defect patterns (reported separately).

**TABLE 1:** Baseline demographics and clinical characteristics of evaluable participants with a confirmed pregnancy outcome (prospective cohort; *N* = 55 pregnancies)

	Prospective cohort (*N* = 55)
**Age at enrollment**	
Mean (SD) years	31.8 (4.73)
Advance maternal age (≥35 years), *n* (%)	17 (31)
**Race, *n* (%)**	
Asian	4 (7)
Black/African American	6 (11)
White/Caucasian	39 (71)
Other^a^	6 (11)
**Trimester of enrollment, *n* (%)**	
First	27 (49)
Second	17 (31)
Third	11 (20)
**Any prenatal testing prior to enrollment, *n* (%)**	
Yes	41 (75)
No	9 (16)
Missing/Unknown	5 (9)
**PGA score (at enrollment), *n* (%)**	
None‐0/mild‐1	20 (36)
Moderate‐2/severe‐3	7 (13)
Missing/unknown	28 (51)
**PGA score (at pregnancy outcome), *n* (%)**	
None‐0/mild‐1	11 (20)
Moderate‐2/severe‐3	3 (5)
Missing/unknown	41 (75)
**SDI cumulative score** ^b^	
*n*	13
Mean (SD)	1.5 (4.68)
Median (IQR)	0 (0, 0)
Min – Max	0–17
**Anti‐dsDNA, *n*** ^c^ (% out of individuals with a result)	
Individuals with test results available	40
*Individuals with at least one abnormal result*	25 (63)
**Complement (C3 or C4), *n*** ^c^ (% out of individuals with a result)	
Individuals with test results available	39
*Individuals with at least one abnormal result*	18 (46)
**aCL, *n*** ^c^ (% out of individuals with a result)	
Individuals with test results available	32
*Individuals with at least one abnormal result*	8 (25)
**LAC, *n*** ^c^ (% out of individuals with a result)	
Individuals with test results available	30
*Individuals with at least one abnormal result*	6 (20)
**Anti‐Ro/SSA and anti‐La/SSB, *n*** ^c^ (% out of individuals with a result)	
Individuals with test results available	32
*Individuals with at least one abnormal result*	15 (47)
**Proteinuria, *n*** ^c^ (% out of individuals with a result)	
Individuals with test results available	39
*Individuals with at least one abnormal result*	10 (26)
**Serum creatinine level (mg/dl)** ^d^	
*n*	72
Mean (SD)	0.72 (0.57)
Median	0.60
Min – Max	0.4–5.3
**Comorbidities and pregnancy complications, *n* (%)**	
Severe lupus flare, pulse steroids required	11 (20)
Hypertensive‐hypertension	10 (18)
Hypothyroidism	9 (16)
Obesity^f^	9 (16)
Thrombocytopenia	6 (11)
Neurological manifestations of lupus^e^	3 (5)
Thrombotic event(s)	2 (4)
Placental abruption	2 (4)
Diabetes type II	2 (4)
Hyperthyroidism	1 (2)
Pregnancy‐associated hypertension	1 (2)
**Previous pregnancy outcomes, *n* (%)**	***N* = 23 previous pregnancies**
Live birth	19 (83)
Pre‐term birth	4 (17)
Miscarriage	7 (30)
Elective termination	3 (13)
Ectopic pregnancy	1 (4)
Birth defects^g^	1 (4)

Abbreviations: aCL, anticardiolipin antibodies; BDE, birth defect evaluator; BMI, body mass index; CVS, chorionic villus sampling; IQR, interquartile range; LAC, lupus anticoagulant; PGA, Physician Global Assessment; SD, standard deviation; SDI, Systemic Lupus International Collaborating Clinics/American College of Rheumatology (SLICC/ACR) Damage Index.

^a^ American Indian/Alaskan Native, Native Hawaiian/Other Pacific Islander, other, and multi‐racial;

^b^ SDI is assessed at enrollment, but data may be obtained at any registry time point;

^c^ at preconception and/or during pregnancy;

^d^ n and summary statistics are across all serum creatinine results, and an individual patient may have contributed 0, 1, 2, or more results;

^e^ that is, psychosis, and seizures;

^f^ BMI ≥30 kg/m^2^ pre‐pregnancy;

^g^ not reviewed or confirmed by the BDE.

### 3.2 Demographic, baseline, and clinical characteristics (prospective cohort)

Baseline demographic and disease characteristics of the 55 prospective cohort individuals with a confirmed pregnancy outcome are provided in Table 1. The mean (standard deviation [SD]) age was 31.8 (4.73) years, and most were White/Caucasian (*n* = 39, 71%). Approximately half of the cohort enrolled in the registry after their first trimester of pregnancy. More than half of the Physician Global Assessment (PGA) scores for SLE severity were missing or unknown at enrollment (28/55; 51%); 20/55 (36%) had none/mild disease activity and 7/55 (13%) had moderate/severe disease activity. Similar results were observed for PGA scores at the time of pregnancy outcome. Systemic Lupus International Collaborating Clinics/American College of Rheumatology (SLICC/ACR) Damage Index (SDI) data were available for only 13/55 (24%) individuals, with a mean score of 1.5 (SD: 4.68; median: 0; range: 0–17), although the timing of SDI completion could not be confirmed.

The most frequent SLE laboratory test available in preconception and/or during pregnancy was anti‐dsDNA, with 25 of the 40 (63%) individuals with at least one available lab result having an abnormal result, followed by complement (*n* = 18/39, 46%) and proteinuria (*n* = 10/39, 26%). Other laboratory tests included anticardiolipin antibodies (aCL) (*n* = 8/32, 25%), anti‐Ro/SSA and anti‐La/SSB (*n* = 15/32, 47%), and lupus anticoagulant (LAC) (*n* = 6/30, 20%); antinuclear antibodies were not included within the study data collection. The mean (SD) serum creatinine level across 72 tests collected during pregnancy was 0.72 (0.57) mg/dl for this cohort (Table 1).

The most common comorbidity or pregnancy complication was severe lupus flare (*n* = 11/55, 20%), and the next most common comorbidity was pre‐existing hypertensive‐hypertension (*n* = 10/55, 18%). Other complications included hypothyroidism (*n* = 9/55, 16%), obesity (*n* = 9/55, 16%), and thrombocytopenia (*n* = 6/55, 11%) (Table 1). Of 23/55 (42%) patients with previous pregnancies, at least one previous miscarriage was reported by seven (*n* = 7/23, 30%) individuals; one (*n* = 1/23, 4%) individual reported a previous child with a birth defect (Table 1).

### 3.3 Belimumab and other exposures during pregnancy (prospective cohort)

For most individuals (*n* = 52/55, 95%), the earliest date of belimumab exposure was prior to conception (Table 2). The last belimumab exposure included first trimester (*n* = 9/55, 16%), second trimester (*n* = 27/55, 49%), third trimester (*n* = 1/55, 2%), and post‐pregnancy outcome (*n* = 18/55, 33%). Mean (SD) cumulative belimumab exposure was 252.8 (114.9) days, and the majority (*n* = 49/55, 89%) received belimumab via the IV route (Table 2). The most common concomitant medications reported during pregnancy were antimalarials (*n* = 45/55, 82%), corticosteroids (*n* = 28/55, 51%), folate (*n* = 26/55, 47%), aspirin (*n* = 22/55, 40%), immunosuppressants (*n* = 16/55, 29%), and nonsteroidal anti‐inflammatory drugs (NSAIDs) (*n* = 9/55, 16%) (Table 2). Further data on concomitant medications during pregnancy and other maternal risk factors are reported in Table 2.

**TABLE 2:** Belimumab and other exposures during pregnancy in evaluable participants with confirmed pregnancy outcomes (prospective cohort; *N* = 55 pregnancies)

	Prospective cohort (*N* = 55)
**Earliest belimumab exposure** ^**a**^, ***n* (%)**	
Preconception	52 (95)
First trimester	2 (4)
Second trimester	1 (2)
Third trimester	0
**Latest belimumab exposure** ^**a**^, ***n* (%)**	
Preconception	0
First trimester	9 (16)
Second trimester	27 (49)
Third trimester	1 (2)
Postpartum^b^	18 (33)
**Belimumab exposure by study timepoints, *n* (%)**	
Preconception through first trimester	9 (16)
Preconception through second trimester	26 (47)
Preconception through third trimester	1 (2)
Preconception through post‐pregnancy outcome^b^	16 (29)
First trimester through second trimester	1 (2)
First trimester through post‐pregnancy outcome^b^	1 (2)
Second trimester through post‐pregnancy outcome^b^	1 (2)
**Route of belimumab administration, *n* (%)**	
IV (or unknown route)	49 (89)
SC	4 (7)
Switched route	2 (4)
**Cumulative belimumab exposure, days, mean (SD)**	252.8 (114.9)
**Concomitant medications** ^**c**^ **during pregnancy, *n* (%)**	
Antimalarials^d^	45 (82)
Corticosteroids (for SLE only)	28 (51)
Folate	26 (47)
Aspirin	22 (40)
Any immunosuppressants	16 (29)
Azathioprine	9 (16)
Methotrexate	5 (9)
Mycophenolate	2 (4)
Cyclosporin	1 (2)
Cyclophosphamide	0
Rituximab	0
NSAID	9 (16)
Heparin	6 (11)
Calcium channel blocker	5 (9)
Epilepsy medication	5 (9)
ACE inhibitor	2 (4)
Beta blockers	3 (5)
Angiotensin II receptor blockers	2 (4)
Insulin	2 (4)
**Alcohol use** ^**c**^ **during pregnancy, *n* (%)**	0
**Tobacco** ^**c**^ ^,^ ^**e**^ **use during pregnancy, *n* (%)**	3 (6)
**Recreational drug** ^**c**^ ^,^ ^**f**^ **use during pregnancy, *n* (%)**	1 (2)

Abbreviations: ACE, angiotensin‐converting enzyme; IV, intravenous; NSAID, nonsteroidal anti‐inflammatory drugs; SC, subcutaneous; SD, standard deviation; SLE, systemic lupus erythematosus.

^a^ Exposure is defined as at least one dose during the period of observation (4 months prior to/during pregnancy for commercial belimumab) and does not imply continued use throughout the period of observation. 100 days have been added to last belimumab treatment to account for five half‐lives (20‐day half‐life*five half‐lives of belimumab [Struemper, Chen, & Cai, 2013]).

^b^ Includes postpartum (for live births), post‐elective terminations and post‐pregnancy loss (miscarriages or stillbirths);

^c^ Concomitant medication, alcohol, tobacco, and recreational drug use data were obtained from the SLE treatment prescriber and/or obstetric healthcare professional, or if not available, the participant/other/pediatrician;

^d^ 300 days have been added to last antimalarial treatment to account for five half‐lives (60‐day half‐life*five half‐lives [Krishna & White, 1996]);

^e^ cigarettes, cigars, smokeless;

^f^ heroin, cocaine, marijuana.

### 3.4 Pregnancy and infant outcomes (prospective cohort)

Of the 55 evaluable pregnancies with a confirmed pregnancy outcome, 53 (96.4%) ended in live births (56 live‐born infants, including three sets of twins), and two were miscarriages; 3.6% (2/55; 95% CI 0.0–8.6%, ad hoc analysis) of pregnancies (without elective termination) resulted in pregnancy loss. Of the two miscarriages, earliest belimumab exposure occurred in preconception and latest exposure occurred post‐pregnancy loss; both pregnancies were exposed to antimalarials (throughout pregnancy) and folate (one case throughout pregnancy, one case preconception‐only). One case also reported calcium channel blocker exposure in all trimesters prior to miscarriage and the other case reported corticosteroid and methotrexate use preconception. None had a history of negative pregnancy outcome or pregnancy complications (gestational diabetes, preeclampsia, eclampsia, Hemolysis Elevated Liver Enzymes and Low Platelets [HELLP] syndrome); no antiphospholipid tests (LAC or aCL antibody) or disease severity scores (PGA or SDI) were available for these two cases.

Of the 56 live‐born infants, 40 (71%) were full‐term births and 16 (29%) were pre‐term births; four (7%) were SGA using the INTERGROWTH‐21st criteria (Villar et al., 2014), and 11 (20%) were SGA using the ACOG criteria (Alexander et al., 1996) (Table 3). At the 4‐month follow‐up visit, 46 (*n* = 46/56, 82%) infants had complete data, of which six (*n* = 6/46, 13%) reported at least one infection or fever, and one (*n* = 1/46, 2%) reported at least one developmental milestone delay. At the 12‐month follow‐up visit, 43 (*n* = 43/56, 77%) infants had complete data, of which 14 (*n* = 14/43, 33%) reported at least one infection or fever; developmental milestone delays were observed for gross motor (*n* = 2/43, 5%), fine motor (*n* = 1/43, 2%), social‐emotional (*n* = 1/43, 2%), and language (*n* = 2/43, 5%) (Table 3).

**TABLE 3:** Pregnancy and infant outcomes in evaluable participants with confirmed pregnancy outcomes within the BPR cohort (overall prospective cohort; *N* = 55 pregnancies)

Outcomes	Prospective cohort
**Pregnancy outcomes, *n* (%)**	***N* = 55 pregnancies**
Live birth	53 (96)
Miscarriage	2 (4)
Molar pregnancy	0
Ectopic pregnancy	0
Elective termination	0
Stillbirth	0
**Infant outcomes**	
**At birth, *n* (%)**	***N* = 56 infants**
Pre‐term birth^a^	16 (29)
Neonatal death	0
SGA^b^	4 (7)
SGA^c^	11 (20)
**At 4 months, *n* (%)**	***N* = 46 infants**
Infants with at least one infection or fever^d^	6 (13)
Developmental milestone delayed by type^e^	
Gross motor	1 (2)
Fine motor	0
Cognitive	0
Socio‐emotional	0
Language	0
**At 12 months, *n* (%)**	***N* = 43 infants**
Infants with at least one infection or fever^d^	14 (33)
Developmental milestone delayed by type^e^	
Gross motor	2 (5)
Fine motor	1 (2)
Cognitive	0
Socio‐emotional	1 (2)
Language	2 (5)

Abbreviations: ACOG, American College of Obstetricians and Gynecologists; BPR, Belimumab Pregnancy Registry; INTERGROWTH‐21st, International Fetal and Newborn Growth Consortium for the 21st Century; SGA, small for gestational age.

^a^ Includes infants from two sets of twins;

^b^ INTERGROWTH‐21st criteria;

^c^ ACOG criteria;

^d^ Any infection or fever of “unknown origin” or “of known infectious etiology that occurs through three months of age for an infant”;

^e^ Provided by the pediatrician and combined across gross motor, fine motor, cognitive, socio‐emotional, and language milestones for cases where this information was available.

### 3.5 Birth defect outcomes (prospective cohort)

Among the 53 live birth pregnancies, 10 resulted in a major birth defect as confirmed by the study BDE (10/53; 18.9%, 95% CI 8.3–29.4%, ad hoc analysis) in 10 infants. There were 13 defect events among the 10 infants (three infants had two defects) and are listed in Table 4. Five infants had six defect events that only met the classification criteria of MACDP: one with nondescending testis, one with congenital heart block, one with pelviectasis, one with ankyloglossia, and one with both positional plagiocephaly and positional torticollis. One infant (with a small fenestrated atrial septal defect) met the classification criteria of EUROCAT but was classified as an exclusionary defect by MACDP. Three infants had defect events (bilateral club foot, congenital heart block, positional plagiocephaly, and torticollis) considered to be defects of known cause, and two infants had defect events (nondescending testis and small ventricular septal defect) with insufficient data on defect and/or belimumab exposure window for proper assessment.

**TABLE 4:** Summary of major birth defect events identified within the BPR cohort (*N* = 10 infants)

Reported cases	Birth defect event reported	Classified by MACDP	Classified by EUROCAT	Organ system	Belimumab exposure timing^a^	Additional considerations
1	Bilateral club foot^b^ ^,^ ^c^	Yes	Yes	Musculoskeletal	Preconception to 2nd trimester	**Defect with known cause**. Can occur due to mechanical factors that take place within the pregnancy
2	Nondescending testis	Yes	No	Male reproductive	Preconception to 2nd trimester	**Insufficient data**. Exposure may not have occurred during critical window of development
3	Very mild Ebstein's anomaly of the tricuspid^b^	Yes	Yes	Cardiovascular	Preconception to postpartum	‐‐‐
4	Congenital heart block^b^	Yes	No	Cardiovascular	Preconception to 2nd trimester	**Defect with known cause**. Associated with SLE disease itself (presence of anti‐Ro/SSA and anti‐La/SSB antibodies)
5	Small ventricular septal defect	Yes	Yes	Cardiovascular	Preconception to 2nd trimester	**Insufficient data**. Described as tiny atypical ventricular septal defect and muscular. Size not known
	Congenital hydronephrosis	Yes	Yes	Renal		‐‐‐
6	Low‐lying conus medullaris	Yes	Yes	CNS	Preconception to 2nd trimester	‐‐‐
	Pelviectasis	Yes	No	Renal		‐‐‐
7	Positional plagiocephaly^b^ ^,^ ^d^	Yes	No	Face	Preconception to 1st trimester	**Defect with known cause**. Can occur due to mechanical factors that take place within the pregnancy; twin pregnancy
	Positional torticollis^b^ ^,^ ^d^	Yes	No	Face		**Defect with known cause**. Can occur due to mechanical factors that take place within the pregnancy; twin pregnancy
8	Small fenestrated atrial septal defect	ED	Yes	Cardiovascular	Preconception to 1st trimester	Prenatal testing prior to enrollment with abnormal results
9	Severe Arnold Chiari type II malformation^d^	Yes	Yes	CNS	Preconception to 2nd trimester	Enrolled in 3rd trimester, prenatal testing done prior to enrollment but results unknown
10	Ankyloglossia	Yes	No	Gastrointestinal	1st trimester to postpartum	‐‐

*Note*: Exclusionary defect (ED); single EDs within an infant are not considered to be birth defect cases.

^a^ 100 days have been added to last belimumab treatment to account for five half‐lives (20‐day half‐live*five half‐lives of belimumab [Struemper et al., 2013]). Data do not imply continued use throughout the period of observation. Preconception is defined as 4 months prior to pregnancy for belimumab;

^b^ Patient was positive for anti‐Ro/SSA and anti‐La/SSB antibodies (all noted at preconception);

^c^ Defect detected prenatally by ultrasound or abnormal prenatal screening in two cases;

^d^ Defect noted after pregnancy outcome (during infant follow‐up).

Of the 10 live‐birth pregnancies with major birth defects, the earliest belimumab exposure occurred preconceptionally in nine (90%) individuals (Table 4). Mean (SD) cumulative belimumab exposure was 224.3 (85.8) days, and the majority (*n* = 9/10, 90%) received belimumab via the IV route. For the 10 pregnancies associated with major birth defects, half (*n* = 5/10, 50%) were enrolled within the second trimester (with four [40%] in the first trimester and one [10%] in the third trimester). Nine (*n* = 9/10, 90%) had prenatal testing prior to enrollment of which one had an abnormal result (infant with atrial septal defect) and one where results were not available (Arnold Chiari type II malformation) (Table 4). All 10 (100%) pregnancies had prenatal testing results post‐enrollment, of which two defects (bilateral club foot and congenital heart block) were detected prenatally and one was suspected (hydronephrosis) by ultrasound or abnormal prenatal screening post‐enrollment. Six (*n* = 6/10, 60%) were of advanced maternal age (35 to 39 years), one (*n* = 1/10, 10%) with a multiple gestation, and one (*n* = 1/10, 10%) reported an elective termination in a previous pregnancy. Of the 10 cases, seven (70%) had anti‐Ro/SSA and anti‐La/SSB tests available (four positive), seven (70%) had aCL tests available (three positive), five (50%) had LAC tests available (two positive), nine (90%) had complement tests available (four abnormal), and nine (90%) had proteinuria tests available (one abnormal).

Of the 10 live‐birth pregnancies associated with major birth defects, six (60%) pregnancies had the presence of at least one comorbidity and/or pregnancy complication, including severe lupus flare, pre‐existing hypertension, hypothyroidism, thrombocytopenia, thrombotic event, and placental abruption. Lupus nephritis was noted in one individual within the BDE case assessment. Three (30%) infants from the 10 live‐birth pregnancies were a pre‐term birth and two were SGA.

Exposure to concomitant medications of interest during pregnancy in the 10 live‐birth pregnancies included antimalarials (*n* = 9/10, 90%), corticosteroids (*n* = 6/10, 60%), aspirin (*n* = 3/10, 30%), azathioprine (*n* = 2/10, 20%), heparin (*n* = 2/10, 20%), antiepileptics (*n* = 1/10, 10%), cyclosporin (*n* = 1/10, 10%); exposure of these drugs occurred in all trimesters. Other concomitant medication exposure included NSAIDs (*n* = 2/10, 20%; one all trimesters, and one in the third trimester) and methotrexate (*n* = 1/10, 10%, at first trimester). Tobacco use was reported during pregnancy for two (20%) of the 10 pregnancies associated with a major birth defect and recreational drug use during pregnancy only for one (10%) case.

### 3.6 Summary of the retrospective cohort

The mean time from pregnancy outcome to enrollment for the 10 individuals in the retrospective cohort was 9 months (range: 7 days to 31 months). The mean (SD) age was 31.1 (4.82) years, the majority (*n* = 8/10, 80%) were White/Caucasian, with a mean (SD) cumulative belimumab exposure of 206.7 (92.2) days. The earliest exposure to belimumab occurred preconceptionally in eight (*n* = 8/10, 80%) individuals. The 10 pregnancies resulted in four (*n* = 4/10, 40%) live births, four (*n* = 4/10, 40%) miscarriages, and two (*n* = 2/10, 20%) elective terminations. No major birth defects were reported for any of the pregnancies and infants in this cohort. Of the four live births, two (*n* = 2/4, 50%) were pre‐term, two (*n* = 2/4, 50%) had at least one infection or fever at the 12‐month follow‐up, and none reported any developmental milestone delay throughout the first year after birth.

Of the four miscarriages, earliest belimumab exposure occurred in preconception for three (75%) cases and first trimester for one case (25%) and latest exposure occurred post pregnancy outcome for all four. Concomitant medication reported during pregnancy within the four miscarriages included corticosteroids (*n* = 3/4, 75%, exposure in all trimesters), NSAID (*n* = 1/4, 25%, all trimesters), antimalarials (*n* = 1/4, 25%, all trimesters), beta blocker (*n* = 1/4, 25%, first trimester), aspirin (*n* = 1/4, 25%, all trimesters), folate (*n* = 1/4, 25%, all trimesters), methotrexate (*n* = 1/4, 25%, first trimester), mycophenolate (*n* = 1/4, 25%, first trimester), and cyclosporin (*n* = 1/4, 25%, all trimesters). Two (*n* = 2/4, 50%) cases had antiphospholipid tests available (aCL and LAC results were unknown for one case, and second case was aCL negative and LAC positive); two (*n* = 2/4, 50%) cases had previous miscarriages while none had a history of pregnancy complications (gestational diabetes, preeclampsia, eclampsia, HELLP syndrome).

## 4 DISCUSSION

The BPR study aimed to evaluate pregnancy outcomes following belimumab exposure. There are currently limited data on the use of belimumab during pregnancy and, although enrollment is low, the BPR provides descriptive data that adds to the overall body of information available. Importantly, this study did not identify multiple defects of a common nature or type that would suggest an unusual pattern or common mechanism of birth defects in those exposed to belimumab.

The exclusion of pregnant individuals from SLE clinical trials and the caution recommended by treatment guidelines (Andreoli et al., 2017; Flint et al., 2016; Sammaritano et al., 2020) may have contributed to the limited data available regarding the use of belimumab in pregnancy. However, a small number of pregnancies have been reported during Phase 2–4 belimumab clinical trials. In an analysis of clinical trial data of the 33 live births, three were associated with congenital abnormalities (one case each of Dandy Walker syndrome, bilateral enlarged kidneys [in a pregnancy exposed to Ambrisentan, a known teratogenic drug], and an unbalanced chromosomal translocation linked to the mother) (Powell, Hill, Eudy, Landy, & Petri, 2014). Furthermore, in a recent real‐world evaluation of 13 patients who received belimumab during pregnancy in Taiwan, 11 resulted in live births and there were no reported fetal anomalies or cases of leukopenia, lymphopenia, neutropenia, or thrombocytopenia (Kao et al., 2021). A single episode of omphalitis was reported, but the fetus recovered following treatment. These patients had a median age of 38 and reported a median of two belimumab courses; almost half also had a history of recurrent pregnancy loss (Kao et al., 2021). Similarly, no major birth defects were observed in an analysis of 12 live births that resulted from 13 pregnancies in patients with SLE who were exposed to belimumab from across three Italian centers (Crisafulli et al., 2021). Additional reports of belimumab exposure during pregnancy have been published as case studies, detailing successful outcomes of three belimumab‐exposed pregnancies, with no reported defects (Chehab et al., 2019; Danve, Perry, & Deodhar, 2014; Emmi et al., 2016; Kumthekar, Danve, & Deodhar, 2017), as well as providing further details on the BPR case with mild Ebstein's anomaly that was also included in our study (Danve et al., 2014). From the review of all available data including the BPR, no multiple defects of a common nature or type were identified that would suggest a pattern or common mechanism of birth defects in individuals receiving belimumab.

In this study, major birth defects according to MACDP and/or EUROCAT criteria were reported in 10 infants in the prospective cohort with a confirmed pregnancy outcome. However, most cases contained factors other than belimumab exposure that can contribute to the presence of birth defects. For example, one patient was receiving methotrexate, which has been associated with birth defects (American College of Rheumatology, 2020; Food and Drug Administration, 2020; Knight & Nelson‐Piercy, 2017; Østensen & Förger, 2013; Taulaigo et al., 2021). Although the proportion of pregnancies resulting in a major birth defect was higher than previously reported for an SLE cohort (18 vs. 8%) (Wallenius et al., 2014), this estimate should be interpreted with caution as the study did not have sufficient sample size (target sample size: *N* = 500 pregnancies) to provide estimates with adequate precision. This is evident with the wide confidence interval provided for the main outcome estimates.

Among the 10 major birth defect cases observed, six (60%) had other explanatory factors. Three defects events from two cases were considered to be defects of known cause by the BDE (bilateral club foot, positional plagiocephaly, and torticollis), as these may occur due to mechanical factors during the pregnancy (Moh, Graham, Wadhawan, & Sanchez‐Lara, 2012; Sharon‐Weiner et al., 2017). One case of nondescending testis may not have been exposed to belimumab during the critical window of development. Congenital heart block, which was observed in one case in this study, is known to be associated with SLE and anti‐Ro/SSA and anti‐La/SBB antibodies (Buyon et al., 2015; Lateef & Petri, 2013), the presence of which had been confirmed in this participant by laboratory testing. There were missing defect details (e.g., septal size) that are required to properly classify as a major defect for the case with ventricular septal defect. Additionally, there is no reason to predict an IgG antibody would affect interventricular septum development (which completes by seven weeks in humans) because belimumab is highly specific for BLyS, which binds to receptors primarily localized to B lymphocytes, and because there is very little placental transfer of IgG antibodies during the first trimester (Dhanantwari et al., 2009; Simister, 2003). Lastly, there was misalignment of defect classifications between MACDP and EUROCAT in five defect events from five cases.

The enrollment target of 500 pregnant individuals for this registry was not met, and only 68 individuals with evaluable data were enrolled, which was not adequate to fully assess study outcomes and provide meaningful results. Recruitment and retention in pregnancy registries is known to be challenging, for reasons including voluntary enrollment, and medication being new to market or infrequently prescribed to individuals trying to conceive or during pregnancy (Food and Drug Administration, 2019; Krueger et al., 2017; Sinclair et al., 2014). In the wider context of pregnancy registries, an analysis of pregnancy registry enrollments, the median pre‐specified target enrollment (among those reporting a target) was 300 (Gelperin et al., 2017). Bird et al. (2018) reported that of 59 products with pregnancy exposure registries, only nine of these enrolled 300 or more patients; the time periods in which these studies achieved the sample size was not provided in the study (Bird et al., 2018). An additional limitation was the exposure‐only design of the BPR, and lack of an internal unexposed SLE comparison group. Comparisons with the general population would not be ideal as SLE is associated with an increased risk of adverse pregnancy outcomes (Bundhun et al., 2017; Marder, 2019; Tani et al., 2021; Wallenius et al., 2014). For example, a meta‐analysis demonstrated there was a significantly higher risk of premature birth (Risk ratio [RR]: 3.05, 95% CI: 2.56–3.63; *p* = .00001), congenital defects (RR: 2.63, 95% CI: 1.93–3.58; *p* = .00001) and stillbirth (RR: 1.70, 95% CI: 1.34–2.16; *p* = .0001) in mothers with SLE compared with those without SLE (Bundhun et al., 2017).

Missing data was an additional limitation that prevented a complete assessment of clinically relevant variables. As previously mentioned, the BPR did not have access to medical records but rather relied on the clinician updating a case record form; it is therefore possible that recall bias impacted these data. Additionally, important missing data included assessment of SLE disease severity and laboratory data. For SLE disease severity, it is possible that severity indices are not routinely used in clinical practice and are thus not available from medical records. For future studies, it would be advisable to build in a component within the study design to prospectively collect this information. Pregnancies in patients with SLE are considered high risk and a higher level of surveillance is undertaken to screen for complications (Lateef & Petri, 2013). Due to medication exposures, risk for congenital heart block with anti‐Ro/SSA, and risk of fetal growth restriction, comprehensive ultrasounds for anatomy and antenatal surveillance may be performed more routinely in this population compared with the lower risk general population (Lateef & Petri, 2013). Therefore, minor birth defects are more likely to be identified in pregnant women with SLE—particularly in a registry setting—than in the general population without SLE, limiting comparisons of defect rates between these two populations.

Despite the BPR failing to meet the recruitment target, collection of data on the use of belimumab in pregnancy remains important, particularly as SLE predominantly affects women of childbearing age (Naseri et al., 2018). To this end, alternative approaches are being implemented to ensure relevant data become available in a timely manner to help inform patients and HCPs. Another approach is the use of existing ongoing infrastructure, such as the Organization of Teratology Information Specialists (OTIS) network of MotherToBaby services (Chambers, Johnson, & Kiernan, 2018; MotherToBaby, 2021), which may be able to recruit at a higher rate due to the existing network. Pregnancy studies within OTIS also have a maternal questionnaire built into the study design to obtain more information on disease severity than was collected within the BPR. Additionally, pregnancy studies conducted within the OTIS network could also provide a concurrent SLE comparison group to improve data generalizability and interpretability, as published data suggests an increased risk of adverse pregnancy outcomes within the SLE population compared with the general population (Barnabe, Faris, & Quan, 2011; Liu et al., 2012; Nili et al., 2013; Vinet et al., 2012; Vinet et al., 2015; Wallenius et al., 2014).

In summary, considering the limitations associated with the BPR, it is not possible to draw conclusions about any relationship between belimumab exposure and major birth defects using these data alone. However, there is no evidence of multiple defects of a common nature or type that suggest a pattern or common mechanism of birth defects within the data reported here or when observed in the wider context of limited case reports.

## FUNDING INFORMATION

The Belimumab Pregnancy Registry (GSK Study BEL114256; NCT01532310) was funded by GSK.

## CONFLICT OF INTEREST

Patricia Juliao, Keele Wurst, Kim Gemzoe, Rebecca Marino, Andrew Liu, and Paige Meizlik are employees of GSK and hold shares in the company. At the time of the study, Jeanne M. Pimenta was an employee of GSK and held shares in the company. At the time of the study, Jennifer Gilbride was an employee of GSK and continues to hold shares in the company. Michelle Petri, R K. Miller, and M. Anthony Moody are members of the BPR Scientific Advisory Committee. Helain Landy has no conflicts of interest to declare. Hugh Tilson is a member of the BPRA Board, and a GSK Retiree. Deborah Covington is an employee of Evidera, a PPD company contracted by GSK to conduct the BPR. Tammy Moore is an employee of PPD the company contracted by GSK to conduct the BPR.

## Data Availability

Information on GSK's data sharing commitments and requesting access can be found at www.clinicalstudydatarequest.com.

## References

[bdr22091-bib-0001] Alexander, G. R. , Himes, J. H. , Kaufman, R. B. , Mor, J. , & Kogan, M. (1996). A United States national reference for fetal growth. Obstetrics and Gynecology, 87(2), 163–168. 10.1016/0029-7844(95)00386-x 8559516

[bdr22091-bib-0002] American College of Rheumatology . (2020). Guideline for the management of reproductive health in rheumatic and musculoskeletal diseases. Supplementary Appendix, 12, 529‐556. 10.1002/art.41191 32090480

[bdr22091-bib-0003] Andreoli, L. , Bertsias, G. K. , Agmon‐Levin, N. , Brown, S. , Cervera, R. , Costedoat‐Chalumeau, N. , … Tincani, A. (2017). EULAR recommendations for women's health and the management of family planning, assisted reproduction, pregnancy and menopause in patients with systemic lupus erythematosus and/or antiphospholipid syndrome. Annals of the Rheumatic Diseases, 76(3), 476–485. 10.1136/annrheumdis-2016-209770 27457513 PMC5446003

[bdr22091-bib-0004] Auyeung‐Kim, D. J. , Devalaraja, M. N. , Migone, T. S. , Cai, W. , & Chellman, G. J. (2009). Developmental and peri‐postnatal study in cynomolgus monkeys with belimumab, a monoclonal antibody directed against B‐lymphocyte stimulator. Reproductive Toxicology, 28(4), 443–455. 10.1016/j.reprotox.2009.07.002 19631735

[bdr22091-bib-0005] Baker, K. P. , Edwards, B. M. , Main, S. H. , Choi, G. H. , Wager, R. E. , Halpern, W. G. , … Albert, V. R. (2003). Generation and characterization of LymphoStat‐B, a human monoclonal antibody that antagonizes the bioactivities of B lymphocyte stimulator. Arthritis and Rheumatism, 48(11), 3253–3265. 10.1002/art.11299 14613291

[bdr22091-bib-0006] Barnabe, C. , Faris, P. D. , & Quan, H. (2011). Canadian pregnancy outcomes in rheumatoid arthritis and systemic lupus erythematosus. International Journal of Rheumatology, 2011, 345727. 10.1155/2011/345727 22028718 PMC3199043

[bdr22091-bib-0007] Bird, S. T. , Gelperin, K. , Taylor, L. , Sahin, L. , Hammad, H. , Andrade, S. E. , … Hampp, C. (2018). Enrollment and retention in 34 United States pregnancy registries contrasted with the Manufacturer's capture of spontaneous reports for exposed pregnancies. Drug Safety, 41(1), 87–94. 10.1007/s40264-017-0591-5 28840499 PMC8979755

[bdr22091-bib-0008] Bundhun, P. K. , Soogund, M. Z. , & Huang, F. (2017). Impact of systemic lupus erythematosus on maternal and fetal outcomes following pregnancy: A meta‐analysis of studies published between years 2001‐2016. Journal of Autoimmunity, 79, 17–27. 10.1016/j.jaut.2017.02.009 28256367

[bdr22091-bib-0009] Buyon, J. P. , Kim, M. Y. , Guerra, M. M. , Laskin, C. A. , Petri, M. , Lockshin, M. D. , … Salmon, J. E. (2015). Predictors of pregnancy outcomes in patients with lupus: A cohort study. Annals of Internal Medicine, 163(3), 153–163. 10.7326/M14-2235 26098843 PMC5113288

[bdr22091-bib-0010] Centers for Disease Control and Prevention . (2019). Metropolitan atlanta congenital defects program. Retrieved from https://www.cdc.gov/ncbddd/birthdefects/macdp.html#Reports

[bdr22091-bib-0011] Chakravarty, E. F. , Nelson, L. , & Krishnan, E. (2006). Obstetric hospitalizations in the United States for women with systemic lupus erythematosus and rheumatoid arthritis. Arthritis and Rheumatism, 54(3), 899–907. 16508972 10.1002/art.21663

[bdr22091-bib-0012] Chambers, C. , Johnson, D. L. , & Kiernan, E. (2018). Approach to evaluating pregnancy safety of anti‐rheumatic medications in the OTIS MotherToBaby pregnancy studies: What have we learned? Rheumatology, 57(suppl_5), v34–v39. 10.1093/rheumatology/key081 30137588 PMC6099128

[bdr22091-bib-0013] Chehab, G. , Krussel, J. , Fehm, T. , Fischer‐Betz, R. , Schneider, M. , Germeyer, A. , … Liebenthron, J. (2019). Successful conception in a 34‐year‐old lupus patient following spontaneous pregnancy after autotransplantation of cryopreserved ovarian tissue. Lupus, 28(5), 675–680. 10.1177/0961203319839482 30907296 PMC6515711

[bdr22091-bib-0014] Clowse, M. E. , Jamison, M. , Myers, E. , & James, A. H. (2008). A national study of the complications of lupus in pregnancy. Am J Obstet Gynecol, 199(2), 127.e1–127.e6. 10.1016/j.ajog.2008.03.012 PMC2542836 18456233

[bdr22091-bib-0015] Correa, A. , Cragan, J. D. , Kucik, J. E. , Alverson, C. J. , Gilboa, S. M. , Balakrishnan, R. , … Chitra, J. (2007). Reporting birth defects surveillance data 1968‐2003. Birth Defects Research. Part A, Clinical and Molecular Teratology, 79(2), 65–186. 10.1002/bdra.20350 17278144

[bdr22091-bib-0016] Crisafulli, F. , Gerardi, M. C. , Moschetti, L. , Fredi, M. , Nalli, C. , Urban, M. L. , … Tincani, A. (2021). POS0702 pregnancy in sle patients treated with belimumab: experience from 3 italian centers. Annals of the rheumatic diseases, 80(Suppl 1), 600–601. 10.1136/annrheumdis-2021-eular.2862

[bdr22091-bib-0017] Danve, A. , Perry, L. , & Deodhar, A. (2014). Use of belimumab throughout pregnancy to treat active systemic lupus erythematosus: A case report. Seminars in Arthritis and Rheumatism, 44(2), 195–197. 10.1016/j.semarthrit.2014.05.006 25005336

[bdr22091-bib-0018] Dhanantwari, P. , Lee, E. , Krishnan, A. , Samtani, R. , Yamada, S. , Anderson, S. , … Lo, C. W. (2009). Human cardiac development in the first trimester: A high‐resolution magnetic resonance imaging and episcopic fluorescence image capture atlas. Circulation, 120(4), 343–351. 10.1161/CIRCULATIONAHA.108.796698 19635979 PMC3411176

[bdr22091-bib-0019] Emmi, G. , Silvestri, E. , Squatrito, D. , Mecacci, F. , Ciampalini, A. , Emmi, L. , & Prisco, D. (2016). Favorable pregnancy outcome in a patient with systemic lupus erythematosus treated with belimumab: A confirmation report. Seminars in Arthritis and Rheumatism, 45(6), e26–e27. 10.1016/j.semarthrit.2016.03.005 27079761

[bdr22091-bib-0020] EUROCAT . (2021) Prevalence charts and tables. Retrieved from https://eu-rd-platform.jrc.ec.europa.eu/eurocat/eurocat-data/prevalence_en

[bdr22091-bib-0021] European Medicines Agency . (2021). ANNEX I ‐ summary of product characteristics, Benlysta. Retrieved from https://www.ema.europa.eu/en/documents/product‐information/benlysta‐epar‐product‐information_en.pdf

[bdr22091-bib-0022] Flint, J. , Panchal, S. , Hurrell, A. , van de Venne, M. , Gayed, M. , Schreiber, K. , … Giles, I. (2016). BSR and BHPR guideline on prescribing drugs in pregnancy and breastfeeding‐part I: Standard and biologic disease modifying anti‐rheumatic drugs and corticosteroids. Rheumatology (Oxford), 55(9), 1693–1697. 10.1093/rheumatology/kev404 26750124

[bdr22091-bib-0023] Food and Drug Administration . (2019). Postapproval pregnancy safety studies: Guidance for industry. Retrieved from https://www.fda.gov/media/124746/download

[bdr22091-bib-0024] Food and Drug Administration . (2020). FDA recommends avoiding use of NSAIDs in pregnancy at 20 weeks or later because they can result in low amniotic fluid. Retrieved from https://www.fda.gov/media/142967/download

[bdr22091-bib-0025] Furie, R. , Petri, M. , Zamani, O. , Cervera, R. , Wallace, D. J. , Tegzová, D. , … van Vollenhoven, R. F. (2011). A phase III, randomized, placebo‐controlled study of belimumab, a monoclonal antibody that inhibits B lymphocyte stimulator, in patients with systemic lupus erythematosus. Arthritis and Rheumatism, 63(12), 3918–3930. 10.1002/art.30613 22127708 PMC5007058

[bdr22091-bib-0026] Gelperin, K. , Hammad, H. , Leishear, K. , Bird, S. T. , Taylor, L. , Hampp, C. , & Sahin, L. (2017). A systematic review of pregnancy exposure registries: Examination of protocol‐specified pregnancy outcomes, target sample size, and comparator selection. Pharmacoepidemiology and Drug Safety, 26(2), 208–214. 10.1002/pds.4150 28028914

[bdr22091-bib-0027] GSK . (2022). Benlysta prescribing information. Retrieved from https://gskpro.com/content/dam/global/hcpportal/en_US/Prescribing_Information/Benlysta/pdf/BENLYSTA-PI-MG-IFU.PDF

[bdr22091-bib-0028] Kao, J. H. , Lan, T. Y. , Lu, C. H. , Cheng, C. F. , Huang, Y. M. , Shen, C. Y. , & Hsieh, S. C. (2021). Pregnancy outcomes in patients treated with belimumab: Report from real‐world experience. Seminars in Arthritis and Rheumatism, 51(5), 963–968. 10.1016/j.semarthrit.2021.06.005 34403811

[bdr22091-bib-0029] Knight, C. L. , & Nelson‐Piercy, C. (2017). Management of systemic lupus erythematosus during pregnancy: Challenges and solutions. Open Access Rheumatology: Research and Reviews, 9, 37–53. 10.2147/oarrr.s87828 28331377 PMC5354538

[bdr22091-bib-0030] Krishna, S. , & White, N. J. (1996). Pharmacokinetics of quinine, chloroquine and amodiaquine. Clinical implications. Clinical Pharmacokinetics, 30(4), 263–299. 10.2165/00003088-199630040-00002 8983859

[bdr22091-bib-0031] Krueger, W. S. , Anthony, M. S. , Saltus, C. W. , Margulis, A. V. , Rivero‐Ferrer, E. , Monz, B. , … Andrews, E. (2017). Evaluating the safety of medication exposures during pregnancy: A case study of study designs and data sources in multiple sclerosis. Drugs Real World Outcomes, 4(3), 139–149. 10.1007/s40801-017-0114-9 28756575 PMC5567459

[bdr22091-bib-0032] Kumthekar, A. , Danve, A. , & Deodhar, A. (2017). Use of Belimumab throughout 2 consecutive pregnancies in a patient with systemic lupus erythematosus. The Journal of Rheumatology, 44(9), 1416–1417. 10.3899/jrheum.170327 28864669

[bdr22091-bib-0033] Lateef, A. , & Petri, M. (2013). Managing lupus patients during pregnancy. Best Practice & Research. Clinical Rheumatology, 27(3), 435–447. 10.1016/j.berh.2013.07.005 24238698 PMC3834352

[bdr22091-bib-0034] Liu, J. , Zhao, Y. , Song, Y. , Zhang, W. , Bian, X. , Yang, J. , … Zhang, F. (2012). Pregnancy in women with systemic lupus erythematosus: A retrospective study of 111 pregnancies in Chinese women. The Journal of Maternal‐Fetal & Neonatal Medicine, 25(3), 261–266. 10.3109/14767058.2011.572310 21504337

[bdr22091-bib-0035] Marder, W. (2019). Update on pregnancy complications in systemic lupus erythematosus. Current Opinion in Rheumatology, 31(6), 650–658. 10.1097/bor.0000000000000651 31464707

[bdr22091-bib-0036] Moh, W. , Graham, J. M. , Wadhawan, I. , & Sanchez‐Lara, P. A. (2012). Extrinsic factors influencing fetal deformations and intrauterine growth restriction. Journal of Pregnancy, 2012, 750485. 10.1155/2012/750485 22888434 PMC3409542

[bdr22091-bib-0037] MotherToBaby . (2021). MotherToBaby pregnancy studies. Retrieved from https://mothertobaby.org/pregnancy-studies/

[bdr22091-bib-0038] Naseri, E. P. , Surita, F. G. , Borovac‐Pinheiro, A. , Santos, M. , Appenzeller, S. , & Costallat, L. T. L. (2018). Systemic lupus erythematosus and pregnancy: A single‐center observational study of 69 pregnancies. Revista Brasileira de Ginecologia e Obstetrícia, 40(10), 587–592. 10.1055/s-0038-1672136 30352455 PMC10316885

[bdr22091-bib-0039] Navarra, S. V. , Guzmán, R. M. , Gallacher, A. E. , Hall, S. , Levy, R. A. , Jimenez, R. E. , … Petri, M. A. (2011). Efficacy and safety of belimumab in patients with active systemic lupus erythematosus: A randomised, placebo‐controlled, phase 3 trial. Lancet, 377(9767), 721–731. 10.1016/s0140-6736(10)61354-2 21296403

[bdr22091-bib-0040] Nili, F. , McLeod, L. , O'Connell, C. , Sutton, E. , & McMillan, D. (2013). Maternal and neonatal outcomes in pregnancies complicated by systemic lupus erythematosus: A population‐based study. Journal of Obstetrics and Gynaecology Canada, 35(4), 323–328. 10.1016/s1701-2163(15)30959-2 23660039

[bdr22091-bib-0041] Østensen, M. , & Förger, F. (2013). How safe are anti‐rheumatic drugs during pregnancy? Current Opinion in Pharmacology, 13(3), 470–475. 10.1016/j.coph.2013.03.004 23522967

[bdr22091-bib-0042] Petri, M. (2020). Pregnancy and systemic lupus erythematosus. Best Practice & Research. Clinical Obstetrics & Gynaecology, 64, 24–30. 10.1016/j.bpobgyn.2019.09.002 31677989

[bdr22091-bib-0043] Powell, M. , Hill, D. , Eudy, A. , Landy, H. , & Petri, M. (2014). Pregnancy outcomes for systemic lupus erythematosus (SLE) subjects with conception during belimumab intravenous (IV) and subcutaneous (SC) placebo‐controlled clinical trials and long term extension trials. Annals of the Rheumatic Diseases, 73, 75.3–75.7576.23912798

[bdr22091-bib-0044] Rowaiee, R. K. (2019). Congenital heart defects in children born to women with systemic lupus erythematosus in Dubai, a nested case‐control study pf Ajch patient records between 2015‐2017. Online journal of cardiovascular research, 1(3). 10.33552/ojcr.2019.01.000511

[bdr22091-bib-0045] Ruiz‐Irastorza, G. , & Khamashta, M. A. (2011). Lupus and pregnancy: Integrating clues from the bench and bedside. European Journal of Clinical Investigation, 41(6), 672–678. 21158850 10.1111/j.1365-2362.2010.02443.x

[bdr22091-bib-0046] Sammaritano, L. R. , Bermas, B. L. , Chakravarty, E. E. , Chambers, C. , Clowse, M. E. B. , Lockshin, M. D. , … D'Anci, K. E. (2020). 2020 American college of rheumatology guideline for the management of reproductive health in rheumatic and musculoskeletal diseases. Arthritis & Rhematology, 72(4), 529–556. 10.1002/art.41191 32090480

[bdr22091-bib-0047] Sharon‐Weiner, M. , Sukenik‐Halevy, R. , Tepper, R. , Fishman, A. , Biron‐Shental, T. , & Markovitch, O. (2017). Diagnostic accuracy, work‐up, and outcomes of pregnancies with clubfoot detected by prenatal sonography. Prenatal Diagnosis, 37(8), 754–763. 10.1002/pd.5077 28568170

[bdr22091-bib-0048] Simister, N. E. (2003). Placental transport of immunoglobulin G. Vaccine, 21(24), 3365–3369. 10.1016/s0264-410x(03)00334-7 12850341

[bdr22091-bib-0049] Sinclair, S. , Cunnington, M. , Messenheimer, J. , Weil, J. , Cragan, J. , Lowensohn, R. , … Tennis, P. (2014). Advantages and problems with pregnancy registries: Observations and surprises throughout the life of the international lamotrigine pregnancy registry. Pharmacoepidemiology and Drug Safety, 23(8), 779–786. 10.1002/pds.3659 24974947 PMC4406353

[bdr22091-bib-0050] Stohl, W. , Schwarting, A. , Okada, M. , Scheinberg, M. , Doria, A. , Hammer, A. E. , … Gordon, D. (2017). Efficacy and safety of subcutaneous Belimumab in systemic lupus erythematosus: A fifty‐two‐week randomized, double‐blind, placebo‐controlled study. Arthritis & Rhematology, 69(5), 1016–1027. 10.1002/art.40049 PMC5434872 28118533

[bdr22091-bib-0051] Struemper, H. , Chen, C. , & Cai, W. (2013). Population pharmacokinetics of belimumab following intravenous administration in patients with systemic lupus erythematosus. Journal of Clinical Pharmacology, 53(7), 711–720. 10.1002/jcph.104 23681782

[bdr22091-bib-0052] Tani, C. , Zucchi, D. , Haase, I. , Larosa, M. , Crisafulli, F. , Strigini, F. A. L. , … Mosca, M. (2021). Are remission and low disease activity state ideal targets for pregnancy planning in systemic lupus erythematosus? A Multicentre Study. Rheumatology, 60, 5610–5619. 10.1093/rheumatology/keab155 33590843

[bdr22091-bib-0053] Taulaigo, A. V. , Moschetti, L. , Ganhão, S. , Gerardi, M.‐C. , Franceschini, F. , Tincani, A. , & Andreoli, L. (2021). Safety considerations when using drugs in pregnant patients with systemic lupus erythematosus. Expert Opinion on Drug Safety, 20, 1–14. 10.1080/14740338.2021.1893298 33599570

[bdr22091-bib-0054] Tsokos, G. C. (2011). Systemic lupus erythematosus. The New England Journal of Medicine, 365(22), 2110–2121. 10.1056/NEJMra1100359 22129255

[bdr22091-bib-0055] Villar, J. , Cheikh Ismail, L. , Victora, C. G. , Ohuma, E. O. , Bertino, E. , Altman, D. G. , … Kennedy, S. H. (2014). International standards for newborn weight, length, and head circumference by gestational age and sex: The newborn cross‐sectional study of the INTERGROWTH‐21st project. Lancet, 384(9946), 857–868. 10.1016/s0140-6736(14)60932-6 25209487

[bdr22091-bib-0056] Vinet, E. , Pineau, C. A. , Clarke, A. E. , Kaouache, M. , Gordon, C. , Platt, R. , & Bernatsky, S. (2012). Major congenital anomalies in children born to women with systemic lupus erythematosus. Arthritis Research & Therapy, 14(Suppl 3), A11. 10.1186/ar3945

[bdr22091-bib-0057] Vinet, E. , Pineau, C. A. , Scott, S. , Clarke, A. E. , Platt, R. W. , & Bernatsky, S. (2015). Increased congenital heart defects in children born to women with systemic lupus erythematosus: Results from the offspring of systemic lupus erythematosus mothers registry study. Circulation, 131(2), 149–156. 10.1161/CIRCULATIONAHA.114.010027 25355915

[bdr22091-bib-0058] Wallenius, M. , Salvesen, K. , Daltveit, A. K. , & Skomsvoll, J. F. (2014). Systemic lupus erythematosus and outcomes in first and subsequent births based on data from a national birth registry. Arthritis Care Res (Hoboken), 66(11), 1718–1724. 10.1002/acr.22373 24839126

[bdr22091-bib-0059] Zhang, F. , Bae, S. C. , Bass, D. , Chu, M. , Egginton, S. , Gordon, D. , … Tanaka, Y. (2018). A pivotal phase III, randomised, placebo‐controlled study of belimumab in patients with systemic lupus erythematosus located in China, Japan and South Korea. Annals of the Rheumatic Diseases, 77(3), 355–363. 10.1136/annrheumdis-2017-211631 29295825 PMC5867402

